# Neuromuscular Response to High-Velocity, Low-Amplitude Spinal Manipulation—An Overview

**DOI:** 10.3390/medicina61020187

**Published:** 2025-01-22

**Authors:** Murdi S. Alanazi, Brian Degenhardt, Gwyn Kelley-Franklin, James M. Cox, Laura Lipke, William R. Reed

**Affiliations:** 1 Rehabilitation Science Program, University of Alabama at Birmingham, Birmingham, AL 35294, USA; 2 Department of Physical Therapy and Health Rehabilitation, College of Applied Medical Sciences, Jouf University, Sakaka 72388, Saudi Arabia; 3 A.T. Still Research Institute, A.T. Still University, Mesa, AZ 85206, USAllipke@binghamton.edu (L.L.); 4 Department of Osteopathic Manipulative Medicine, Kirksville College of Osteopathic Medicine, A.T. Still University, Kirksville, MO 63501, USA; 5 Independent Researcher, Private Practice, Fort Wayne, IN 46805, USA; 6 Department of Physical Therapy, University of Alabama at Birmingham, Birmingham, AL 35294, USA

**Keywords:** manual therapy, neuromuscular, high-velocity, low-amplitude spinal manipulation, spinal manipulation, electromyography

## Abstract

The clinical use of spinal manipulation to treat musculoskeletal conditions has nearly tripled in the United States since 1980, and it is currently recommended by most global clinical guidelines as a conservative treatment for musculoskeletal pain, despite a lack of knowledge concerning its mechanisms of action. This overview highlights evidence of direct neuromuscular responses to high-velocity, low-amplitude spinal manipulation (HVLA-SM) as delivered by chiropractic, osteopathic, and physical therapy clinicians, with an intent to foster greater interprofessional dialogue and collaborative research to better address current gaps in mechanistic knowledge of the neuromuscular response to HVLA-SM. Three databases (PubMed, CINAHL Ultimate (EBSCO), EMBASE (Elsevier)) were searched from 2000 to December 2024 with specific search terms related to thrust HVLA-SM and the neuromuscular response. To focus strictly on neuromuscular responses related to HVLA-SM, this literature overview excluded articles using non-HVLA-SM manual therapy techniques (i.e., massage, non-thrust joint mobilization, and/or combined HVLA-SM with other forms of treatment such as exercise or non-thrust joint mobilization) and studies in which patient-centered outcomes (i.e., pain scores) were the primary outcomes of the HVLA-SM interventions. Pediatric studies, animal studies, and studies in languages other than English were also excluded. One-hundred and thirty six articles were identified and included in this overview. Neuromuscular findings related to HVLA-SM in the areas of electromyography (EMG), muscle thickness, muscle strength, reflexes, electroencephalogram (EEG), and evoked potential were often mixed; however, evidence is beginning to accumulate either in favor of or opposed to particular neuromuscular responses to HVLA-SM as larger and more scientifically rigorous studies are being performed. Recurrent limitations of many HVLA-SM-related studies are small sample sizes, leading to a lack of generalizability, and the non-standardization of HVLA-SM delivery, which has prevented researchers from arriving at definitive conclusions regarding neuromuscular responses to HVLA-SM. Discussions of future neuromuscular research needs related to HVLA-SM are included for clinicians and researchers inside and outside of the field of manual therapy, to advance this field.

## 1. Introduction

High-velocity, low-amplitude spinal manipulation (HVLA-SM) is one of the oldest manual therapy interventions in clinical use today [1]. HVLA-SM’s popularity as a nonpharmacological treatment for musculoskeletal pain is increasing worldwide, despite the current lack of understanding regarding its underlying physiological mechanisms, though this limits its appropriate clinical use and overall clinical efficacy. HVLA-SM is most often delivered by chiropractic, osteopathic, and physical therapy clinicians and is used primarily to treat musculoskeletal pain, joint immobility, and/or muscular hypertonicity/spasm. HVLA-SM is often defined as a technique that involves delivering a short-duration thrust (typically <150 ms) of low amplitude, in which a joint or vertebra is carried just beyond its normal physiological range of movement without exceeding the boundaries of anatomic integrity [2]. In recent years, multiple review articles involving HVLA-SM and various neurophysiological, biomechanical, or autonomic effects have been published, often with a specific emphasis on either the anatomical location of the delivered HVLA-SM (i.e., cervical, thoracic, or lumbar spine), a specific clinical condition (i.e., lower back pain, neck pain, etc.), and/or neurophysiological or autonomic responses [3,4,5,6,7,8,9,10,11,12,13,14,15,16,17]. Despite an increasing amount of HVLA-SM research having been conducted within the last two decades, our mechanistic knowledge related to HVLA-SM remains extremely limited. Few, if any, HVLA-SM related systematic reviews are able to draw definitive conclusions about HVLA-SM treatment due to their insufficient levels of investigation and/or the mixed evidence, which is largely the result of inadequate study designs, small sample sizes, lack of appropriate controls, and/or lack of HVLA-SM delivery standardization. However, progress is beginning to be made in addressing several of these limitations. Keeping informed about the most recent HVLA-SM neuromuscular research findings will help to better inform future investigations into the underlying physiological mechanisms of HVLA-SM, while encouraging the development of new interdisciplinary and inter-professional research collaborations around this growing non-pharmacological approach to healthcare.

## 2. Methods

**Figure 1:**
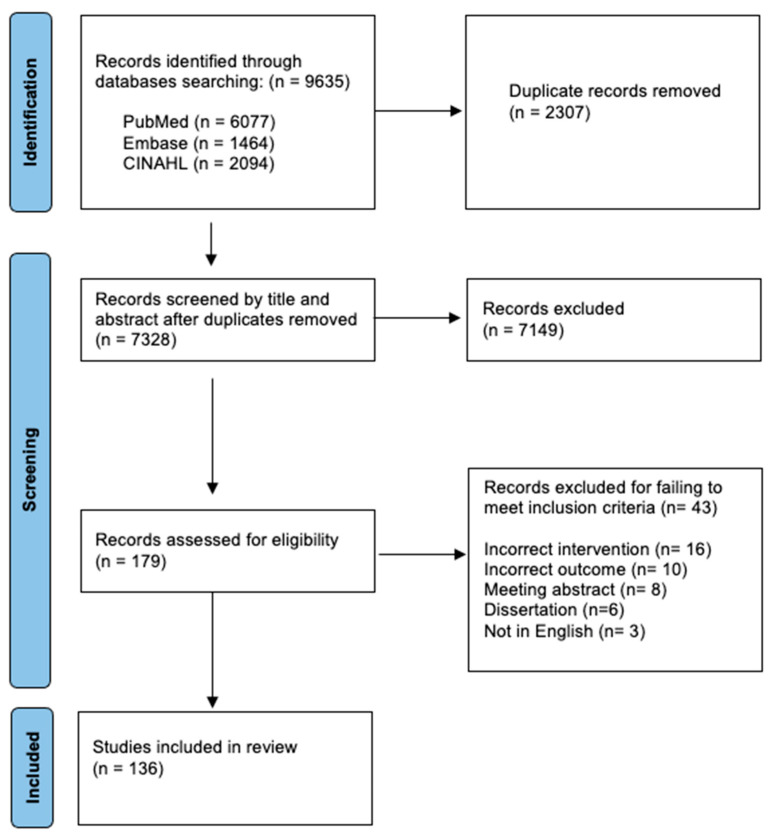
Flowchart diagram.

This overview highlighting the neuromuscular response to HVLA-SM is the result of International Consortium of Manual Therapies (ICMT) clinicians’ and researchers’ joint efforts to foster greater interprofessional dialogue and to further encourage greater interdisciplinary and interprofessional collaborative research investigating the physiological mechanisms underlying HVLA-SM. A systematic search of the PubMed database was completed in July 2024, and that search was recently updated (December 2024) and expanded to include a systematic search of the PubMed, CINAHL Ultimate (EBSCO), and EMBASE (Elsevier) databases from 2000 to December 2024 with the assistance of a research librarian (LL). The full search strategies, utilizing a comprehensive combination of keywords and controlled vocabulary, were translated for each database. The exact strategies are provided in Supplementary Materials File S1. For this overview, searches were limited to peer-reviewed studies published between 2000 and 2025 meeting the inclusion criteria of (a) original research; (b) human studies (excluding pediatrics and case studies); (c) published in English; and (d) using neuromuscular measures directly related to HVLA-SM alone and not in combination with any other therapeutic interventions. The results from each database were uploaded to PaperPile and Covidence for deduplication and screening (Figure 1). All screening was completed by two independent reviewers (MA and WR). Disputes were resolved through discussion and mutual agreement. A total of 136 studies were incorporated into the overview (Figure 1). Search results were provided to ICMT team members (MA, BD, GF, JC, LL, WR) via PaperPile Reference Manager software (version: 1.5.797), in categorized subfolders organized by two team members (GF, BD). Evaluation of the quality and rigor of individual HVLA-SM-related studies included in this overview was beyond the scope of the current work; however, future studies can seek to address this aspect of HVLA-SM research.

## 3. Results

### 3.1. Muscle/Tendon Mechanoreceptors

Changes in the muscle spindle response and stretch sensitivity have long been theorized to play a mechanistic role in the clinical efficacy of HVLA-SM [18,19]. Muscle spindle afferents lie parallel to extrafusal muscle fibers and convey mechanical-related information (changes in muscle length and velocity/rate) to the central nervous system, which is important for proprioception and movement [20]. Due to the invasive nature of recording direct muscle spindle responses, most muscle spindle evidence related to HVLA-SM comes from animal studies, which were not included in this overview. Lima et al. [21] recently reviewed and summarized muscle spindle findings of these animal studies, as it relates to HVLA-SM. Muscle spindles and Golgi tendon organs (GTOs) are key mechanoreceptors involved in joint position sense and proprioception, and thus are potentially involved in mechanisms of HVLA-SM. However, only a small number of human studies have investigated the effects of HVLA-SM on joint position sense or proprioception. HVLA-SM has been shown in a limited number of studies to improve cervical [22], elbow [23], and ankle [24] joint position sense. However, in asymptomatic, chronic lower back pain, a single session of HVLA-SM (one to four thrusts) failed to produce an immediate or lasting impact in various proprioceptive measures (joint position sense, threshold to detect passive motion, direction of motion, and/or force reproduction) [25], with a similar lack of findings in neck pain when individuals received cervical [26] or thoracic [27] manipulation. Global pelvic manipulation also failed to produce changes in knee joint position sense at 30 and 60 degrees compared to a control group [28], whereas lumbopelvic manipulation in individuals with patellofemoral pain improved knee joint position sense at 60 degrees but not at 20 degrees [29].

### 3.2. Muscle Activation (EMG) and Conduction Velocity

Electromyography (EMG) is a technique used to evaluate and record electrical activity produced by skeletal muscle. The amplitude and timing of EMG signals are frequently used to quantify changes in static and dynamic muscle activation following HVLA-SM and other forms of manual therapy [30,31,32]. Trunk EMG responses to varied dosage/force–time profiles of HVLA-SM in humans have been investigated [32,33,34,35,36,37,38,39,40]. Greater thrust magnitudes elicited higher trunk EMG responses [33,40], with device-delivered post-HVLA-SM responses ranging from 2.4 to 18.1 ms in duration [33]. EMG responses typically occur between 0 and 400 ms after the onset of a manual HVLA-SM thrust and last for ~100 to 400 ms afterwards [41,42]. An increase in the muscle response delay (50–340 ms) occurred with an increase in HVLA-SM impulse duration (100 to 1500 ms), indicating distinctive muscle response differences between shorter-duration thrust HVLA-SM maneuvers and longer spinal mobilization maneuvers [42]. This finding was confirmed in a subsequent muscle activation study comparing HVLA-SM to spinal mobilization [43]. Increasing the preload force prior to the HVLA-SM thrust decreased the trunk EMG response during and after HVLA-SM [38], and upon the delivery of two HVLA-SMs in quick succession, the peak EMG, peak force, and rate were found to be higher following the second thrust in both asymptomatic and symptomatic participants [44].

Both manual and device-delivered cervical/thoracolumbar HVLA-SM elicit local and distant muscle activity, but often not uniformly across participants at distant sites [31,45,46,47,48]. In individuals with mild neck disability, the highest rate of EMG response following cervical or thoracic HVLA-SM was localized in nature [47], supporting similar findings reported in other studies [46,49]. However, while the greatest EMG activation levels may occur in close proximity to the HVLA-SM delivery site, distant EMG responses in asymptomatic participants were frequently recorded in the lumbar musculature following cervical HVLA-SM [45]. Use of surface (erector spinae) and indwelling (multifidus) electrodes also demonstrated trends of decreasing EMG muscle response rates and increasing muscle-activity-onset delays as the distance from the HVLA-SM delivery site increased [46]. A linear relationship was demonstrated between applied peak HVLA-SM forces and paraspinal EMG activation [32]. Furthermore, a positive linear relationship was also reported between distant EMG changes in the bilateral biceps brachii muscles in healthy individuals following right-sided cervical (C5–6) HVLA-SM, with 94.2% and 80.04% EMG increases on the right and left arms, respectively, where the right-sided EMG was significantly greater than the left [50]. This bilateral increase in biceps EMG activity occurred irrespective of cervical facet joint cavitation [50]. Increases in deltoid EMG activity also occurred following cervical HVLA-SM during subsequent prolonged (30 s) isometric contractions, without differences during short (5 s) isometric or isotonic contractions [51]. Thoracic HVLA-SM elicited widespread increases in shoulder muscle EMG activity during arm elevation in individuals with subacromial pain syndrome, but this study lacked a control group [52]. Immediate bilateral increases in transversus abdominis/internal oblique muscle activation activity were also demonstrated after sacroiliac manipulation during rapid voluntary arm movement, suggesting improved segmental stability in the motor response [53]. HVLA-SM increased the EMG activity of oblique abdominal muscles, but not that of the deep trunk transversus muscles (recorded with intramuscular fine-wire electrodes) or the rectus abdominis or anterior deltoid muscle (recorded with surface electrodes), in individuals with lower back pain [54]. The authors of that particular study noted high variability between individuals, which is consistent with the results reported in several other EMG studies [55,56].

In contrast to the aforementioned EMG studies demonstrating increases in muscle activity following HVLA-SM, an acute reduction in trunk or extremity EMG activity is also commonly reported following HVLA-SM [30,56,57,58,59,60,61,62,63]. Some studies even report a lack of recorded muscle activation post-HVLA-SM altogether [30,31,42,59,60,64,65,66,67,68,69,70,71,72]. Additional studies demonstrating a lack of change in EMG activity post-HVLA-SM include the following: no changes in upper-extremity EMG activity (deltoid and biceps brachii muscles) reported after a C5 HVLA-SM in neck pain patients [73]; and thoracic HVLA-SM failing to significantly alter trapezius and posterior deltoid EMG activity during treadmill walking when compared to a sham control group [74]. Lumbopelvic manipulation failed to alter EMG activity of the vastus medialis and gluteus medius in individuals with patellofemoral pain syndrome [75]. Assessment of indwelling and surface EMG trunk activity delays in asymptomatic and symptomatic lower-back-pain individuals after HVLA-SM revealed a non-significant decrease in EMG response in the symptomatic group, as well as a mean delay across all active muscles in the symptomatic group of 14 ms greater than in the asymptomatic group [41]. A significant reduction in deep trunk EMG response was also reported after a trunk muscle counterstrain intervention (median decrease of 3.3%) procedure, but not after thrust HVLA-SM [64]. Other studies have similarly reported no between-group EMG changes following HVLA-SM [76,77,78].

EMG studies have also investigated potential changes in muscle activity during functional movements performed immediately following HVLA-SM. For example, upon performing dynamic flexion/extension movements after HVLA-SM, decreases [58,79,80,81] or lack of change [58,65,79,82,83,84,85] in EMG activity are reported. Decreases in EMG activity after HVLA-SM can be a result of either decreased drive to alpha motor units or increased alpha motor inhibition, with the latter currently being viewed as more favorable [59,60,86], particularly considering the abnormally elevated trunk EMG activity demonstrated during resting conditions of individuals with lower back pain [86,87,88,89]. Dynamic extension-related EMG decreases after HVLA-SM are in contrast to a lower-back-pain study in which the maximal isometric extension EMG activity increased after HVLA-SM [40]. In this particular isometric extension study, it was uncertain whether a decline in lower back pain led to a reduction in self-imposed effort and inhibition, or whether increased excitability of the motor neuron pool was responsible for the increases in EMG activity [40].

In a study using intramuscular EMG, the average muscle conduction velocity (CV) of the tibialis anterior muscle increased after HVLA-SM in the isometric steady state and during ramp contractions (10% MVC) compared to a control exhibiting no change in discharge rate [90]. This increase in CV could be explained by the recruitment of new motor units [90]. However, these intramuscular EMG findings are in contrast to high-density surface EMG findings used to evaluate CV changes in ankle dorsiflexion at two force levels and patterns (5% and 10% MVC during “ramp” and “ramp and maintain” force production). This later study found a significant reduction in CV post-HVLA-SM only after the 5% MVC “ramp and maintain” condition, and no CV changes at 10% MVC [91]. Differences in study findings may be related to differences in methodology, inherent data variability, and/or differences in HVLA-SM delivery. Another study reported that L4/L5 HVLA-SM increased CV related to the soleus T-reflex compared to sham manipulation [92]. Again, the lack of standardized HVLA-SM delivery, small sample sizes, and other methodological differences are likely great contributors to these mixed EMG results post-HVLA-SM.

### 3.3. Muscle Strength

Short-term increases in muscle strength following HVLA-SM have been reported in a variety of muscles and patient populations [40,90,93,94,95,96,97,98,99,100,101,102,103,104,105,106,107,108]. These increases in muscle strength are attributed, at least partially, to increases in cortical drive [97,99,104,109,110]. Increases in lower trapezius strength following thoracic HVLA-SM were reported in healthy individuals [93]. Handgrip strength increased in judo athletes and non-athletes following cervical or thoracic HVLA-SM [94,111]. Pain-free handgrip strength in the arm affected by lateral epicondylalgia increased after cervical HVLA-SM [96], but no increase in handgrip strength was reported in a separate lateral epicondylalgia study [106] or in an asymptomatic population [112,113]. Increases in lower limb muscle strength after HVLA-SM have also been reported in healthy and neurologically impaired individuals [90,97,98,100,101,102,110,114,115]. Among active military personnel, the mean isometric pulling strength significantly increased after 4 weeks of chiropractic care involving HVLA-SM compared to a wait-list control [116]. While the vast majority of the aforementioned studies examined immediate changes in muscle strength post-HVLA-SM, increases in plantar flexor strength were demonstrated at 30 min but not 60 min post-HVLA-SM [99]. No difference in quadriceps maximum voluntary peak force was found after a series of thoraco/lumbar and/or pelvic HVLA-SM treatments in individuals with patellofemoral pain [117]. Three HVLA-SM foot treatments significantly increased vertical jump height in young female athletes with talocrural joint dysfunction [118]. Among axial muscles, increases in mastication muscle maximal bite strength (11% immediately following HVLA-SM and 13% at 1 week) post-HVLA-SM were reported, which to our knowledge is the longest duration of a muscle strength effect reported post-HVLA-SM [119]. Continued study around the enhancement and duration of muscle strength increases after HVLA-SM in different populations is needed, in addition to studies involving large sample sizes.

While an overwhelming majority of the studies report short-term increases in muscle strength immediately post-HVLA-SM, no changes in handgrip strength were shown in recreational basketball players [112], in knee flexion/extension at 5 and 20 min post-HVLA-SM in college-aged healthy adults [120], or in other asymptomatic strength testing studies [121,122,123]. It should be noted that in the aforementioned knee flexion/extension study, a substantial standard deviation of mean peak torque likely contributed to the lack of muscle strength changes following HVLA-SM [120]. In patients with chronic non-specific neck pain, a single cervical and thoracic HVLA-SM was no more effective than the control in inducing immediate changes in grip strength [124].

### 3.4. Muscle Thickness

HVLA-SM also resulted in significant immediate decreases in terminal and global spinal stiffness, while ultrasonic measures indicated that HVLA-SM increased lumbar multifidus recruitment during contraction [125]. A case-series study reported short-term decreases in lateral abdominal muscle (transverse abdominis, internal oblique) thickness while at rest following HVLA-SM, with an increase in transverse abdominis thickness during contraction post-HVLA-SM [126]. However, in larger studies, lumbar HVLA-SM failed to alter transverse abdominis muscle thickness at rest or during contraction [127,128], along with similar failures of changes in muscle thickness reported in transverse abdominis and internal oblique muscles in a subgroup of individuals who met a proposed clinical prediction rule for a lumbar stabilization exercise [129].

### 3.5. M-Wave, H-Reflex, V-Wave, and F-Wave Changes

The electrically evoked direct motor response (M-waves), F-waves, Hoffman reflexes (H-reflexes), and volitional waves (V-waves) are commonly used as indices of spinal presynaptic inhibition and/or motoneuron excitability [130,131,132]. The classical methodology used to evoke H-reflexes consists of stimulating a peripheral nerve at various intensities to obtain recruitment curves, which are used to determine the H/M ratio. H-reflexes can be recorded while at rest or during maximum voluntary contraction (MVC). The V-wave is obtained when a supramaximal stimulus is delivered to a peripheral nerve during an MVC [130] and is a measure of supraspinal input or cortical drive to motor neurons [132]. HVLA-SM elicited no change in M-waves [109] but increased V-wave amplitude [97,98,99] in multiple studies, for which the changes reported lasted at least 60 min post-HVLA-SM. F-waves assess the antidromic activation of a subset of lower motor neurons at the spinal level (i.e., spinal excitability). HVLA-SM resulted in no changes in F-wave parameters [109,133,134]. Multiple studies demonstrate that HVLA-SM decreases H-wave peak amplitude [49,86,98,134,135,136,137,138,139,140,141], thereby indicating a transient decrease in motoneuron excitability or decreases in the threshold required to elicit the H-reflex [98]. However, other studies reported no changes in H-reflex parameters following HVLA-SM [97,99,140,142,143,144]. In a similar study, spinal reflex excitability of the quadriceps in individuals with knee pathology and quadriceps inhibition was reported as not being altered by HVLA-SM of the lumbopelvic region or knee [144].

In several early HVLA-SM-related H-reflex studies, it was suggested that participant movement and/or repositioning artifacts may have been partly or fully responsible for the reported HVLA-SM-related decreases in the H-reflex peak amplitude [140]. However, HVLA-SM treatment of individuals with lower back pain showed a significant reduction in H-reflex responses without any patient repositioning, with baseline recovery within 60 s [145]. This HVLA-SM-related decrease in H-reflex parameters was confirmed in subsequent studies using different methodologies and data analyses [98,139], indicating that movement or position artifacts, while present, are likely not the main contributor to decreased Hmax/Mmax ratios after HVLA-SM [139]. Finally, it should be noted that in a letter to the editor on the general topic of H-reflexes, it was pointed out that the H-reflex is susceptible to inhibition and facilitation from many sources including passive muscle stretching, antagonist contraction, and the state of mind of the participant when tested [146], and this knowledge related to the H-reflex must be kept in mind when considering studies investigating HVLA-SM and H-reflex changes. With regard to other reflex-oriented changes related to HVLA-SM, a recent study reported that 8 weeks of HVLA-SM decreased the cervico-ocular reflex gain compared to a wait-list control in individuals with subclinical neck pain [147], while another study reported no changes in the soleus T-reflex amplitude following L4/L5 HVLA-SM compared to sham manipulation [92].

### 3.6. MEPs/SEPs/CSPs/MRCPs/CAPs and EEG

Changes in motor evoked potential (MEP), cortical silent periods (CSPs), and somatosensory evoked potential (SEP) related to HVLA-SM treatment have just begun to be investigated. MEPs are electrical signals recorded from neural tissue or muscle following the activation of central motor pathways. Single- or repetitive-pulse stimulation over the cortex by transcranial magnetic stimulation (TMS) causes the spinal cord and peripheral muscles to produce MEPs. While highly variable, MEPs are considered a measure of net corticospinal excitability. Differences in MEPs following HVLA-SM are mixed [109,110,133,137,148,149,150,151,152,153]. Fryer et al. reported a decrease in MEP amplitude following a lumbosacral HVLA-SM, whereas Haavik and colleagues reported either increases or no MEP changes with HVLA-SM [110,137,154]. A single HVLA-SM resulted in a significantly larger MEP amplitude recorded from the tibialis posterior compared to a control in a chronic stroke population [110]. A single HVLA-SM resulted in no change in erector spinae MEP amplitude and erector spinae stretch reflex amplitude in patients with LBP; however, the subset of participants experiencing an audible HVLA-SM-initiated response exhibited a 20% decrease in the stretch reflex [155]. When using TMS, MEPs and CSPs were obtained in a population with a history of ankle sprain receiving either foot joint mobilization, thrust manipulation, or a control (hand placement only) [148]. Thrust manipulation increased cortical excitability while joint mobilization treatment decreased it. Only the thrust manipulation group demonstrated a significant increase in the maximal MEP amplitude of the tibialis anterior after the intervention [148]. No CSP changes were found between groups [148]. Transient decreases in CSP duration (up to 20 min) after HVLA-SM were reported [133,154], in addition to CSP increases depending on the muscle involved [133]. A peristimulus time histogram and peristimulus frequency gram analysis indicated that CSPs are decreased by 25.3 ms and 34.7 ms, respectively, post-HVLA-SM [154]. TMS-related I-wave activation was also reported to be increased post-HVLA-SM, suggesting an HVLA-SM-related excitability increase in some low-threshold motor units [154]. These findings need to be confirmed in larger studies.

TMS-related short-interval intracortical inhibition (SICI), short-interval intracortical facilitation (SIFC), and stimulus–response curves (SR) were also evaluated following HVLA-SM [133]. Together, these outcomes are considered to be measures of sensorimotor integration at the cortical level. HVLA-SM decreased SICI in the abductor pollicis brevis and increased SICI in the extensor indicis proprius muscles, while SIFC increased in the abductor pollicus brevis and decreased in the extensor indicis proprius, suggesting mediated inhibition in a motor-specific manner [133]. However, a lack of SICI changes following HVLA-SM and motor sequence learning was reported, while motor sequence learning alone significantly decreased SICI [156]. The plateau of the SR curve (referred to as the MEPmax) was demonstrated to increase for both upper- and lower-limb muscles following HVLA-SM and TMS-induced activity [109,157]. TMS-induced change in cerebellar inhibition related to HVLA-SM was investigated in participants with neck pain [157]. Participants receiving HVLA-SM had a 90% reduction in cerebellar inhibition following a motor acquisition task versus a 50% reduction for healthy participants receiving HVLA-SM, and only a 1% reduction in neck pain participants receiving the sham control [157]. The duration or potential clinical benefit of these cerebellar changes has yet to be determined, but that study suggests that individuals with ongoing neck pain may have impaired sensory integration in the cerebellum and/or primary motor cortex, which is possibly improved by HVLA-SM [157], but additional study is needed. The movement-related cortical potential (MRCP) reflects cortical changes prior to any actual movement initiation; thus, if HVLA-SM elicits cortical changes, then MRCP changes should be evident. MRCP parameters of latency, amplitude of early Bereitschaftspotential (EBP), late Bereitschaftspotential, peak negativity, and the rebound rate of reafferent potential were determined in relation to HVLA-SM. Significant differences post-HVLA-SM were reported for EBP amplitude and peak negativity, with all other MRCP parameters demonstrating no differences [109]. These findings provide additional support for the concept of HVLA-SM altering cortical changes related to motor control processing that are not solely spinal-cord-mediated.

SEPs evaluate the function of the somatosensory pathway from the periphery (i.e., median nerve) to the upper spinal cord or cerebral cortex (as measured from the scalp). Early evidence suggests that HVLA-SM can alter cortical SEP signaling. HVLA-SM caused a 17 to 30% attenuation [158,159,160,161,162,163] in cortical P22-N30 complex SEP peak amplitudes, with N30 being the site of the most significant changes, where declines in some cases lasted 20–30 min post-HVLA-SM [159,161]. These SEP changes occur predominantly in the prefrontal cortex, which is a key sensorimotor integration site for executive functions [158]. In a recent study of young individuals with subclinical neck pain (i.e., ache, stiffness, discomfort), a single HVLA-SM thrust directed towards either a clinically dysfunctional cervical spinal segment or a non-dysfunctional cervical spinal vertebra yielded nearly a 17% decrease in N30 amplitude when HVLA-SM was directed toward a clinically identified dysfunctional cervical vertebra compared to a non-dysfunctional cervical vertebra [163]. This decrease in N30 amplitude was measured immediately after HVLA-SM, leaving the duration of this HVLA-related effect unknown, and these changes may or may not be different from those experiencing moderate to severe neck pain or other neurological impairments. For instance, HVLA-SM significantly reduced N30 amplitude in individuals with Alzheimer’s (15%) [164], but had no effects on N30 amplitude in Parkinson’s individuals [164], while N30 amplitude increased (39%) after HVLA-SM in a chronic stroke population without affecting resting-state EEG in any of these three populations [164,165]. Frontal lobe N30 decreases related to HVLA-SM were observed in multiple studies [153,158,159,160,164], accompanied at times by an attenuation in pain [160]. The N30 site has multiple neural generators, including primary sensory and motor cortices, basal ganglia, thalamus, and premotor areas [166,167], and thus is thought to reflect early sensorimotor integration [167]. Reciprocal sensory inhibition (the filtering of afferent information by the somatosensory system to enhance contrast from stimuli applied to adjacent body parts) might be a possible mechanism for cortical attenuation in SEP amplitudes. HVLA-SM failed to alter SEPs at peripheral or subcortical recording sites [159]. A recent resting-state EEG study indicated a significant increase in functional connectivity between the posterior cingulate cortex and parahippocampal regions following HVLA-SM in subacute to chronic post-stroke individuals [168]. Parietal lobe N20 SEP peak amplitude decreases were also reported in some studies [159], while no N20 changes were observed in other studies [165].

There were no changes in resting-state EEG source localization, delta–alpha ratio, or brain symmetry index following HVLA-SM in a chronic stroke population [165]. A recent resting-state EEG study in participants with mild cognitive impairment indicated a decrease in power for delta and theta bands and an increase in power for the beta2 band immediately after HVLA-SM [169], whereas resting-state EEG in a lower-back-pain population following either a single HVLA-SM or 4 weeks of HVLA-SM treatment indicated an increase in alpha activity between brain regions within the Default Mode Network (active areas of the brain when a person is not paying attention to external or internal stimuli or thinking goal-oriented thoughts) [153]. In an EEG study investigating the effect of audible joint cavitation during a thoracic HVLA-SM, both audible and non-audible HVLA-SM elicited significant changes in brain activity in the frontal, parietal, and occipital lobes, whereas no change in the temporal lobe (audible centers) was reported with joint cavitation [170].

In a study of nine individuals undergoing lumbar decompression surgery, Colloca et al. [171] reported that seventy-five percent of instrument-delivered HVLA-SM resulted in positive compound action potential (CAP) responses (peak–peak response > 2.5× baseline), with a mean latency of 12.0 ms (range 8.2–17.3 ms). Magnitudes of CAP responses were significantly greater for HVLA-SM compared to sham HVLA-SM, and it was noted that greater force magnitudes (>150 N), as typically recorded during manually delivered HVLA-SM, may further increase the amplitude of CAP responses related to HVLA-SM [171].

## 4. Discussion

A greater understanding of superficial and deep sensory and mechanoreceptor responses to HVLA-SM may be important to elucidating its physiological mechanisms. Muscle spindles are only one of many types of sensory receptors stimulated during HVLA-SM delivery, and there remains much work to be carried out to investigate HVLA-SM responses in other types of sensory receptors and how these receptors interact and impact the central nervous system. For example, GTOs convey sensory information regarding the force of muscle contraction, and unlike muscle spindles that are parallel to muscle fibers, GTOs are in series, which can explain GTOs’ increased sensitivity to active (vs. passive) force generation [172]. While it may be possible to excite GTOs with passive stretching during manual therapy treatments, such activation is typically thought to be a result of more aggressive therapeutic interventions and therefore is thought less likely to be a large contributor to mechanoreceptor input related to passively delivered HVLA-SM [173]. However, more research is needed to elucidate the precise relationship between HVLA-SM and these two types of mechanoreceptors, as well as any clinical importance. The current overview clearly illustrates that the majority of clinically relevant questions remain unanswered pertaining to the relationship between the specific HVLA-SM sensory receptor(s) response and its potential influence on central processing, proprioception changes, and/or clinical outcomes. The questions of whether certain thresholds of sensory receptor activation must be passed to elicit observable changes in higher somatosensory centers or to yield more of a sustained physiological response remain unanswered.

Collectively, the research findings appear to indicate that HVLA-SM can sufficiently modify the short-term pattern of muscle activation (EMG) locally, and to a lesser degree distally. However, EMG results related to HVLA-SM still remain somewhat mixed, despite the numerous studies investigating the EMG response. This is most likely a result of methodological differences, small sample sizes, lack of standardization of HVLA-SM delivery, as well as multiple challenges surrounding EMG recordings, including the non-homogeneity of asymptomatic/symptomatic populations; the inherent variability in manually delivered HVLA-SM, the participant posture, and/or the performance of repeated flexion/extension movement tasks; the layer, location, and anatomical side of the muscle studied; and varying aspects of the normalization process, participant motivation, and/or training [46,174]. One must also remain mindful that the potential relationship between HVLA-SM-induced short-term EMG changes and any positive clinical outcomes has yet to be established. HVLA-SM thrust characteristics can and do impact the EMG response, with EMG differences being reported between HVLA-SM and other manual therapy techniques. However, despite HVLA-SM differences in the EMG response versus other forms of manual therapy, no between-group differences were reported for spinal stiffness change and terminal or global spinal stiffness [43,175]. Therefore, the clinical relevance of EMG changes related to HVLA-SM is somewhat questionable and needs further clarification in rigorously designed studies addressing many of the aforementioned limitations inherent to EMG, particularly the standardization of HVLA-SM delivery. It should also be considered that a minimum threshold of applied HVLA-SM force may be required in order to elicit distant changes in EMG muscle activity and/or even result in positive clinical outcomes in diverse neuromuscular conditions treated with HVLA-SM. Along these lines, it is particularly interesting to note that animals with disc degenerative changes showed a 25–30% reduction in multifidus EMG response to HVLA-SM [176], but this finding has yet to be confirmed in humans.

Evidence appears to be mounting with regard to HVLA-SM resulting in short-term increases in muscle strength. These changes in muscle strength are currently attributed to changes in cortical drive, but additional study is needed to confirm and further characterize the peripheral and central effects of HVLA-SM in both asymptomatic and symptomatic populations. It is encouraging to see an increasing number of studies investigating muscle strength changes related to HVLA-SM in neurologically impaired individuals (i.e., stroke, Parkinson’s disease, etc.). These studies hold great promise in revealing HVLA-SM-related central cortical drive changes. To date, only a few studies demonstrate muscle strength changes lasting over 30 min following HVLA-SM, with most studies demonstrating changes in muscle strength post-HVLA-SM lasting 10 min or less. HVLA-SM studies investigating reflex changes also indicate rather transitory changes, making it difficult to demonstrate a direct relationship between these transitory muscle strength and reflex changes and positive clinical outcomes. However, the actual duration of these physiological changes following HVLA-SM may not prove to be a critical factor, as anyone can attest who has ever experienced a momentary electrical power surge or power outage in electronic devices. Reflex-related HVLA-SM results remain very mixed. This is likely due in large part to methodological and/or analytical differences, as well as non-standardized HVLA-SM delivery. It should also be kept in mind that HVLA-SM-related changes in reflex amplitudes appear to be time- and muscle-dependent, as revealed by their functional and adaptive properties [177].

Investigations involving HVLA-SM-related changes in MEPs, SEPs, and EEGs are just emerging and demonstrate great potential in helping to reveal peripheral and cortical mechanisms of HVLA-SM. HVLA-SM-related N20 and N30 SEP peak changes are thought to reflect the processing of peripheral afferent inputs at the primary somatosensory cortex (S1) [178,179] and early sensorimotor integration [167]. Transient decreases in CSP duration after HVLA-SM were reported in several studies, and the cortical inhibitory mechanisms responsible for CSP changes are thought to involve GABAB-mediated intracortical inhibition, inhibitory projection neurons, or a withdrawal of excitatory input to pyramidal cells thanks to increased inhibition of such excitatory pathways [180,181]. While transitory cortical changes related to HVLA-SM form an exciting area of new research, these early cortical studies must be evaluated with some caution due to frequent limitations often encountered in HVLA-SM-related research, such as small sample sizes, limited generalizability, impaired population heterogeneity, inherent technological challenges (i.e., low signal-to-noise ratios, high degrees of signal filtering, and/or interpretations of neural signaling events that are potentially unrelated to the HVLA-SM intervention itself), and lack of standardization of HVLA-SM delivery. As mentioned in other HVLA-SM research areas, only the immediate effects of HVLA-SM on MEPs, SEPs, and EEGs have been investigated to date, and the duration and/or clinical relevancy of these transient changes are presently unknown. A recent systematic review of cortical changes related to HVLA-SM concluded that the early evidence does point to short-term changes, but also noted study design or methodological concerns in several of these early studies in this area [182]. In the coming decade, as technology advances and additional evidence from larger studies involving HVLA-SM-related MEP, SEP, and EEG responses in acute and chronic neuromuscular populations is reported, we will achieve greater clarity on the mechanistic and/or clinical importance of these peripheral and central physiological measures and HVLA-SM.

## 5. Future Directions

Dozens of sensory receptors have long been theorized to be stimulated during HVLA-SM based on the magnitude of applied mechanical forces [183,184,185,186]. While not specifically reviewed in the current work, a sustained line of investigation of the muscle spindle afferent response to simulated HVLA-SM in animal models has demonstrated that physical thrust characteristics of HVLA-SM such as peak load, thrust rate, and anatomical location greatly influence peripheral muscle spindle afferent signaling to the central nervous system during and/or following HVLA-SM in healthy and dysfunctional spinal joints [21,187,188]. A better understanding of the HVLA-SM dosage characteristics, duration of physiological effects, and diminution of applied mechanical HVLA-SM forces due to tissue viscoelasticity will be essential to determining the physiological effects and underlying mechanisms of HVLA-SM. In addition, it should be noted that the physiological and clinical importance of the specific HVLA-SM application site still remains unclear. A systematic review of 10 studies investigating the importance of the HVLA-SM application site (clinically vs. non-clinically relevant sites) did not indicate a superior clinical outcome of one over the other [189], but it is premature for any definitive conclusions to be drawn with so few studies having been conducted on this topic. Fully characterizing HVLA-SM-related changes in EMG, muscle strength, reflexes, cortical activity, central processing, and neuroplasticity is important and will require improved study designs, use of appropriate controls, larger sample sizes, applied force quantification, and better standardization of HVLA-SM delivery. A better understanding of HVLA-SM-related physiological effects in symptomatic (particularly in acute and/or chronic musculoskeletal populations) and neurologically impaired populations will also prove beneficial to advancing the field. In addition, another area requiring future investigation is the contribution of contextual factors related to HVLA-SM delivery and clinical outcomes. These gaps in our HVLA-SM knowledge, along with others, have recently been identified and discussed in greater detail [190,191]. Perhaps one of the greatest challenges continuing to face the field today is demonstrating a relationship between immediate and short-term HVLA-SM-related physiological changes and positive clinical outcomes. This task is made particularly difficult as there is a very limited understanding of all definitive short-duration neuromuscular changes occurring as a result of HVLA-SM treatment. In addition, elucidating the neurophysiological differences between therapeutic touch inherent to all manual therapies and the mechanisms strictly associated with HVLA-SM is also an area in need of greater investigation by investigators inside and outside the field of manual therapy [192].

## 6. Limitations

This overview was not intended to be an exhaustive search of all the available literature involving direct neuromuscular effects of clinical HVLA-SM, as this would entail searches of additional databases from inception, the inclusion of articles in other languages besides English, and a more comprehensive search of the literature using broader terminology that conveys manual therapy treatments equivalent to HVLA-SM but using different verbiage. Each manual therapy profession has developed its own unique terminology, which creates communication and research comprehension difficulties for investigators or clinicians outside of a given manual therapy profession [193]. This overview failed to assess the scientific rigor of the studies cited, and as noted multiple times, the small sample sizes of many of the studies limits the strength and generalizability of the reported findings. These and other limitations will need to be addressed in future studies; however, the primary purpose of the present overview is to encourage greater dialogue and collaboration between clinicians and researchers inside and outside of manual therapy regarding the physiological responses and mechanisms of HVLV-SM.

## 7. Conclusions

HVLA-SM has been demonstrated to elicit multiple short-term physiological effects on sensory receptors, muscle strength, reflexes, EMG, MEP, SEP, and EEG responses. However, determining the clinical relevance of these transient neuromuscular effects remains challenging and requires intentional effort as well as better standardization of HVLA-SM delivery. Identifying the specific physiological mechanisms responsible for the clinical efficacy of HVLA-SM is a somewhat daunting task due to the complexity and the multitude of body systems and tissues that are impacted by HVLA-SM. That said, it is encouraging that the amount of HVLA-SM-related research continues to grow rapidly, holding much promise for the field of manual therapy, though there remains a great need for much more basic and clinical HVLA-SM-related research.

## Data Availability

No new data were created or analyzed in this study. The search terms used for the current study are included in the Supplementary Materials File S1 or are available from the corresponding author on reasonable request.

## Supplementary Materials

The following supporting information can be downloaded at https://www.mdpi.com/article/10.3390/medicina61020187/s1, File S1: Search Term Strategy.
