# Supplementary material for: Neuromuscular Response to High-Velocity, Low-Amplitude Spinal Manipulation—An Overview
Source: Medicina (Kaunas). 2025 Jan 22;61(2):187. doi: 10.3390/medicina61020187 (PMC11857552; doi:10.3390/medicina61020187)
Supplement: Supplementary file 1 [file medicina-61-00187-s001.zip › medicina-3352854-supplementary.pdf]

Supplementary File S1

Database: PubMed
Host: National Library of Medicine
Data Parameters: 2000-2025

"manipulation, osteopathic"[MeSH Terms] OR "osteopathic manipulation"[Title/Abstract] OR "osteopathic treat*"[Title/Abstract] OR "OMT"[Title/Abstract] OR "manipulation, chiropractic"[MeSH Terms] OR "chiropractic manipulation"[Title/Abstract] OR "chiropractic adjustment"[Title/Abstract] OR "chiropractic care"[Title/Abstract] OR "manipulation, orthopedic"[MeSH Terms] OR "thrust*"[All Fields] OR "manipulation, spinal"[MeSH Terms] OR "spinal manipulat*"[All Fields] OR "spine manipulat*"[All Fields] OR "cervical manipulat*"[All Fields] OR "lumbar manipulat*"[All Fields] OR "spinal adjust*"[All Fields] OR "high-velocity low-amplitude"[All Fields] OR "high-velocity"[All Fields] OR "low-amplitude"[All Fields] OR "HLVA"[All Fields] OR "HVLA-SM"[All Fields]

AND

"Muscle Strength"[MeSH Terms] OR "Muscle Strength"[Title/Abstract] OR "Muscle Contraction"[MeSH Terms] OR "Muscle Contraction"[Title/Abstract] OR "maximum voluntary contraction"[Title/Abstract] OR "Mechanoreceptors"[MeSH Terms] OR "mechanoreceptor*"[Title/Abstract] OR "muscle spindle"[Title/Abstract] OR "golgi tendon organs"[Title/Abstract] OR "stretch sensitivity"[Title/Abstract] OR "muscle activ*"[Title/Abstract] OR "muscle conduction velocity"[Title/Abstract] OR "muscle action potential"[Title/Abstract] OR "motor response"[Title/Abstract] OR "action potential"[Title/Abstract] OR "m-wave"[Text Word] OR "volitional wave"[All Fields] OR "v-wave"[Text Word] OR "f-wave"[Text Word] OR "hoffmans reflex*"[Title/Abstract] OR "H-Reflex"[MeSH Terms] OR "h reflex*"[Title] OR "Proprioception"[MeSH Terms] OR "Proprioception"[Title/Abstract] OR "joint position sense"[Title/Abstract] OR "neurophysiolog*"[Title/Abstract] OR "autonomic*"[Title/Abstract] OR "afferent"[Title/Abstract] OR "alpha motor unit*"[Title/Abstract] OR "excitab*"[Title/Abstract] OR "muscle conduction velocity"[Title/Abstract] OR "MVC"[Title/Abstract] OR "resting state"[Title/Abstract] OR "evoked potentials, motor"[MeSH Major Topic] OR "motor evoked potentials"[Title/Abstract] OR "MEP"[Title/Abstract] OR "evoked potentials, somatosensory"[MeSH Major Topic] OR "somatosensory evoked potentials"[Title/Abstract] OR "cortical silent period*"[Title/Abstract] OR "movement-related cortical potentials"[Title/Abstract] OR "MRCP"[Title/Abstract] OR "Electromyography"[MeSH Terms] OR "electromyograph*"[Title/Abstract] OR "Electroencephalography"[MeSH Terms] OR "electroencephalograph*"[Title] OR "EEG"[Title] OR "evoked potentials, motor"[MeSH Terms] OR "evoked motor potential*"[Title/Abstract] OR "evoked potentials, somatosensory"[MeSH Terms] OR "evoked somatosensory potential*"[Title/Abstract] OR "Nerve Conduction Studies"[MeSH Terms] OR "nerve conduction stud*"[Title/Abstract] OR

"amplitude"[Title/Abstract] OR "velocity"[Title/Abstract] OR "latency"[Title/Abstract] OR "Muscle Strength Dynamometer"[MeSH Terms]

NOT (("infant"[mesh] OR "child"[mesh] OR "adolescent"[mesh] OR "Pediatrics"[Mesh]) NOT adult[mesh])

NOT (("Animals"[MESH] OR "Animal Experimentation"[MESH] OR "Models, Animal"[MESH] OR "Vertebrates"[MESH]) NOT ("Humans"[MESH] OR "Human experimentation"[MESH]))

Database: CINAHL Ultimate
Host: EBSCOhost
Data Parameters: 2000-2025

(MH "manipulation, osteopathic+") OR (TI "osteopathic manipulation" OR AB "osteopathic manipulation") OR (TI "osteopathic treat*" OR AB "osteopathic treat*") OR (TI OMT OR AB OMT) OR (MH "manipulation, chiropractic+") OR (TI "chiropractic manipulation" OR AB "chiropractic manipulation") OR (TI "chiropractic adjustment" OR AB "chiropractic adjustment") OR (TI "chiropractic care" OR AB "chiropractic care") OR (MH "manipulation, orthopedic+") OR thrust* OR (MH "manipulation, spinal+") OR "spinal manipulat*" OR "spine manipulat*" OR "cervical manipulat*" OR "lumbar manipulat*" OR "spinal adjust*" OR "high-velocity low-amplitude" OR high-velocity OR low-amplitude OR HLVA OR HVLA-SM

AND

(MH "Muscle Strength+") OR (TI "Muscle Strength" OR AB "Muscle Strength") OR (MH "Muscle Contraction+") OR (TI "Muscle Contraction" OR AB "Muscle Contraction") OR (TI "maximum voluntary contraction" OR AB "maximum voluntary contraction") OR (MH Mechanoreceptors+) OR (TI mechanoreceptor* OR AB mechanoreceptor*) OR (TI "muscle spindle" OR AB "muscle spindle") OR (TI "golgi tendon organs" OR AB "golgi tendon organs") OR (TI "stretch sensitivity" OR AB "stretch sensitivity") OR (TI "muscle activ*" OR AB "muscle activ*") OR (TI "muscle conduction velocity" OR AB "muscle conduction velocity") OR (TI "muscle action potential" OR AB "muscle action potential") OR (TI "motor response" OR AB "motor response") OR (TI "action potential" OR AB "action potential") OR m-wave OR "volitional wave" OR v-wave OR f-wave OR (TI "hoffmans reflex*" OR AB "hoffmans reflex*") OR (MH H-Reflex+) OR (TI "h reflex*") OR (MH Proprioception+) OR (TI Proprioception OR AB Proprioception) OR (TI "joint position sense" OR AB "joint position sense") OR (TI neurophysiolog* OR AB neurophysiolog*) OR (TI autonomic* OR AB autonomic*) OR (TI afferent OR AB afferent) OR (TI "alpha motor unit*" OR AB "alpha motor unit*") OR (TI excitab* OR AB excitab*) OR (TI "muscle conduction velocity" OR AB "muscle conduction velocity") OR (TI MVC OR AB MVC) OR (TI "resting state" OR AB "resting state") OR (MM "evoked potentials, motor+") OR (TI "motor evoked potentials" OR AB "motor evoked

potentials") OR (TI MEP OR AB MEP) OR (MM "evoked potentials, somatosensory+") OR (TI "somatosensory evoked potentials" OR AB "somatosensory evoked potentials") OR (TI "cortical silent period*" OR AB "cortical silent period*") OR (TI "movement-related cortical potentials" OR AB "movement-related cortical potentials") OR (TI MRCP OR AB MRCP) OR (MH Electromyography+) OR (TI electromyograph* OR AB electromyograph*) OR (MH Electroencephalography+) OR (TI electroencephalograph*) OR (TI EEG) OR (MH "evoked potentials, motor+") OR (TI "evoked motor potential*" OR AB "evoked motor potential*") OR (MH "evoked potentials, somatosensory+") OR (TI "evoked somatosensory potential*" OR AB "evoked somatosensory potential*") OR (MH "Nerve Conduction Studies+") OR (TI "nerve conduction stud*" OR AB "nerve conduction stud*") OR (TI amplitude OR AB amplitude) OR (TI velocity OR AB velocity) OR (TI latency OR AB latency) OR (MH "Muscle Strength Dynamometer+")

Database: Embase
Host: Elsevier
Data Parameters: 2000-2025

'osteopathic manipulation'/exp OR 'osteopathic manipulation' OR 'osteopathic manipulation':ti,ab,kw OR 'osteopathic treat*':ti,ab,kw OR 'omt':ti,ab,kw OR 'chiropractic manipulation'/exp OR 'chiropractic manipulation' OR 'chiropractic manipulation':ti,ab,kw OR 'chiropractic adjustment':ti,ab,kw OR 'chiropractic care':ti,ab,kw OR 'orthopedic manipulation'/exp OR 'orthopedic manipulation' OR 'thrust*' OR 'spine manipulation'/exp OR 'spine manipulation' OR 'spinal manipulat*' OR 'spine manipulat*' OR 'cervical manipulat*' OR 'lumbar manipulat*' OR 'spinal adjust*' OR 'high-velocity low-amplitude' OR 'high-velocity' OR 'low-amplitude' OR 'hlva' OR 'hvla-sm'

AND

('muscle strength'/exp OR 'muscle strength' OR 'muscle strength':ti,ab,kw OR 'muscle contraction'/exp OR 'muscle contraction' OR 'muscle contraction':ti,ab,kw OR 'maximum voluntary contraction':ti,ab,kw OR 'mechanoreceptor'/exp OR 'mechanoreceptor' OR 'mechanoreceptor*':ti,ab,kw OR 'muscle spindle':ti,ab,kw OR 'golgi tendon organs':ti,ab,kw OR 'stretch sensitivity':ti,ab,kw OR 'muscle activ*':ti,ab,kw OR 'muscle action potential':ti,ab,kw OR 'motor response':ti,ab,kw OR 'action potential':ti,ab,kw OR 'm-wave':ti,ab,kw,de,dn,df,mn,tn OR 'volitional wave' OR 'v-wave':ti,ab,kw,de,dn,df,mn,tn OR 'f-wave':ti,ab,kw,de,dn,df,mn,tn OR 'hoffmans reflex*':ti,ab,kw OR 'hoffmann reflex'/exp OR 'hoffmann reflex' OR 'h reflex*':ti OR 'proprioception'/exp OR 'proprioception' OR 'proprioception':ti,ab,kw OR 'joint position sense':ti,ab,kw OR 'neurophysiolog*':ti,ab,kw OR 'autonomic*':ti,ab,kw OR 'afferent':ti,ab,kw OR 'alpha motor unit*':ti,ab,kw OR 'excitab*':ti,ab,kw OR 'muscle conduction velocity':ti,ab,kw OR 'mvc':ti,ab,kw OR 'resting state':ti,ab,kw OR 'motor evoked potential'/exp/mj OR 'motor evoked potentials':ti,ab,kw OR 'mep':ti,ab,kw OR 'somatosensory evoked potential'/exp/mj OR 'somatosensory evoked potentials':ti,ab,kw OR 'cortical silent period*':ti,ab,kw OR 'movement-related cortical potentials':ti,ab,kw OR 'mrcp':ti,ab,kw OR 'electromyography'/exp OR 'electromyography'

OR 'electromyograph*':ti,ab,kw OR 'electroencephalography'/exp OR 'electroencephalography' OR 'electroencephalograph*':ti OR 'eeg':ti OR 'motor evoked potential'/exp OR 'motor evoked potential' OR 'evoked motor potential*':ti,ab,kw OR 'somatosensory evoked potential'/exp OR 'somatosensory evoked potential' OR 'evoked somatosensory potential*':ti,ab,kw OR 'electroneurography'/exp OR 'electroneurography' OR 'nerve conduction stud*':ti,ab,kw OR 'amplitude':ti,ab,kw OR 'velocity':ti,ab,kw OR 'latency':ti,ab,kw OR 'dynamometer'/exp OR 'dynamometer') NOT ('animal'/exp OR 'animal' OR 'animal experiment'/exp OR 'animal experiment' OR 'animal model'/exp OR 'animal model' OR 'vertebrate'/exp OR 'vertebrate') NOT ('human'/exp OR 'human' OR 'human experiment'/exp OR 'human experiment') NOT ('infant'/exp OR 'infant' OR 'child'/exp OR 'child' OR 'adolescent'/exp OR 'adolescent' OR 'pediatrics'/exp OR 'pediatrics') NOT ('adult'/exp OR 'adult')
